# Therapeutic targeting of the hepcidin-ferroportin axis and erythropoietic modulators: a narrative review

**DOI:** 10.3389/fmed.2025.1726337

**Published:** 2025-12-09

**Authors:** Ghaith K. Mansour, Ahmad W. Hajjar, Muhammad Raihan Sajid

**Affiliations:** 1 College of Pharmacy, Alfaisal University, Riyadh, Saudi Arabia; 2 College of Medicine, Alfaisal University, Riyadh, Saudi Arabia

**Keywords:** hepcidin, ferroportin, iron metabolism, erythroferrone, ferroptosis, luspatercept, TMPRSS6, hematologic disorders

## Abstract

Iron homeostasis represents a critical regulatory network in hematologic diseases, with dysregulation contributing to diverse pathological conditions ranging from iron-loading anemias to hematologic malignancies. The hepcidin molecule acts as a master regulator and works in conjunction with its sole iron exporter, the ferroportin channel, orchestrating systemic iron balance through intricate molecular mechanisms involving bone morphogenetic protein (BMP) signaling, erythroferrone, and transmembrane serine protease 6 (TMPRSS6). Recent advances have identified ferroptosis, an iron-dependent cell death pathway, as both a pathogenic mechanism and therapeutic target in hematologic disorders. This narrative review synthesizes current understanding of iron regulatory pathways and examines emerging therapeutic approaches targeting hepcidin-ferroportin dysfunction, erythroferrone modulation, and ferroptosis induction. Novel agents, including luspatercept, matriptase-2 inhibitors, and anti-hemojuvelin antibodies, represent promising interventions for conditions characterized by ineffective erythropoiesis and iron maldistribution. The integration of pharmacogenomic approaches and precision medicine strategies offers potential for optimized therapeutic outcomes in iron-related hematologic diseases. Critical evaluation of clinical trial evidence reveals both therapeutic promise and implementation challenges, highlighting the need for continued mechanistic research and translational development. Future directions emphasize combination therapeutic strategies, biomarker-driven patient stratification, and the development of targeted interventions addressing the complex interplay between iron metabolism, inflammation, and hematopoietic function.

## Introduction

Iron homeostasis represents one of the most tightly regulated metabolic pathways in human physiology, with profound implications for hematopoietic function and clinical outcomes in diverse blood disorders (1). The central role of iron in oxygen transport, cellular respiration, and DNA synthesis makes its precise regulation essential for maintaining erythropoietic capacity while preventing the deleterious effects of iron overload (2). Over the past decade, revolutionary advances in understanding iron regulatory mechanisms have unveiled sophisticated molecular networks governing systemic iron balance, opening unprecedented therapeutic opportunities for treating previously intractable hematologic conditions (3). The discovery of hepcidin as the master regulator of iron homeostasis has fundamentally transformed our conceptual framework for understanding iron-related diseases (4). This hepatically-derived peptide hormone controls iron absorption, distribution, and cellular release through its interaction with ferroportin, the sole known cellular iron exporter (5). Dysregulation of the hepcidin-ferroportin axis underlies diverse pathological conditions, from hereditary hemochromatosis characterized by hepcidin deficiency to anemia of chronic inflammation marked by hepcidin excess (6).

Recent molecular discoveries have revealed additional layers of complexity in iron regulation, including the erythroid regulator erythroferrone (ERFE), which suppresses hepcidin during periods of increased erythropoietic demand (1). The transmembrane serine protease matriptase-2 (TMPRSS6) has emerged as a crucial negative regulator of hepcidin expression, with mutations causing iron-refractory iron deficiency anemia (IRIDA) through inappropriate hepcidin elevation (7). While the core components of iron homeostasis are well-established, the rapid translation of this knowledge into novel therapeutics has created a dynamic and complex landscape. Existing reviews often focus on individual pathways, such as the hepcidin-ferroportin axis or ferroptosis in isolation. This narrative review aims to synthesize these interconnected areas, providing a comprehensive overview of the current therapeutic pipeline targeting iron metabolism in hematologic diseases. We will critically evaluate emerging agents–from hepcidin modulators and erythroid maturators to ferroptosis inducers–placing clinical trial data within the context of mechanistic understanding and practical clinical challenges. Furthermore, we will identify key controversies, such as the long-term implications of hepcidin suppression, and discuss how precision medicine approaches are poised to optimize the use of these sophisticated therapies. This review will provide a consolidated, critical perspective on how targeting iron biology is reshaping treatment paradigms across a spectrum of hematologic disorders.

## The hepcidin-ferroportin regulatory axis

Hepcidin functions as the master regulator of iron homeostasis through its direct interaction with ferroportin, the ubiquitous cellular iron exporter expressed in duodenal enterocytes, macrophages, and hepatocytes (8). Upon binding to ferroportin, hepcidin induces rapid internalization and proteasomal degradation of the iron exporter, effectively blocking iron release from cells into the circulation (9). This regulatory mechanism represents a sophisticated homeostatic system that responds to multiple physiological stimuli, including iron status, inflammation, and demand for erythropoiesis (10). Structural studies have revealed that hepcidin binding to ferroportin is coupled to iron binding, with an 80-fold increase in hepcidin affinity in the presence of iron (9). This iron-dependent binding mechanism ensures that only iron-loaded ferroportin molecules are targeted for degradation, providing precise control over iron export activity (11). The renal hepcidin-ferroportin axis has been shown to control iron reabsorption and determine the magnitude of kidney and systemic iron overload (12).

Hepcidin expression is regulated by multiple signaling pathways, with bone morphogenetic protein 6 (BMP6) serving as the primary iron-sensing mechanism (13). The BMP6-SMAD signaling cascade, operating through the co-receptor hemojuvelin, provides iron-responsive control of hepcidin transcription (14). Inflammatory stimuli, particularly interleukin-6, represent another major regulatory input that can override iron-sensing mechanisms, leading to anemia of chronic disease (ACD) (15).

### Erythroferrone and erythropoietic regulation

Erythroferrone (ERFE) represents a critical erythroid-derived hormone that suppresses hepcidin expression during periods of increased erythropoietic activity (1). This glycoprotein is secreted by erythroid precursors in response to erythropoietin stimulation and functions as a physiological antagonist of BMP6-mediated hepcidin induction (16). ERFE deficiency results in inappropriate hepcidin elevation and impaired iron mobilization for erythropoiesis, demonstrating its essential role in coordinating iron availability with erythropoietic demand (1).

The therapeutic potential of targeting ERFE has been demonstrated through the development of anti-ERFE antibodies that prevent hepcidin suppression and ameliorate murine thalassemia by specifically targeting the N-terminal domain of erythroferrone (ERFE) (16). These findings highlight the critical role of ERFE in iron-erythropoiesis coordination and suggest novel therapeutic approaches for conditions characterized by ineffective erythropoiesis (1).

### TMPRSS6 and hepcidin suppression

Matriptase-2 (TMPRSS6) functions as a crucial negative regulator of hepcidin expression. Its established mechanism involves the proteolytic cleavage of membrane-bound hemojuvelin (HJV), the BMP6 co-receptor, thereby dampening the BMP-SMAD signaling cascade and subsequent hepcidin transcription (17, 18). This role is highlighted by mutations in TMPRSS6 that cause iron-refractory iron deficiency anemia (IRIDA) due to inappropriately elevated hepcidin (19). However, the understanding of TMPRSS6’s function is evolving. Recent studies suggest a more complex model where matriptase-2 also regulates iron homeostasis by setting the basal levels of hepatic hepcidin expression through a non-proteolytic mechanism that may involve the formation of a complex with HJV and neogenin-1, independently of its enzymatic activity (20). This does not necessarily contradict the proteolytic model but rather adds a layer of regulation. It appears that TMPRSS6 employs both proteolytic and non-proteolytic means to fine-tune hepcidin production, ensuring it is appropriately suppressed under iron-deficient conditions. The relative contributions of these mechanisms in different physiological contexts remain an active area of investigation (20–22). The intricate regulation of the hepcidin-ferroportin axis, ERFE, and TMPRSS6 underscores the precision of systemic iron control. However, dysregulation at any of these mechanisms can precipitate disease, creating a compelling rationale for therapeutic intervention. The following sections explore how this deep mechanistic understanding has been translated into a diverse portfolio of investigational and approved drugs designed to correct iron dysregulation at its source.

## Therapeutic targeting of iron regulatory pathways

### Hepcidin agonists and antagonists

The central role of hepcidin in iron regulation has made it an attractive therapeutic target for both iron overload and iron-restricted conditions (23). Hepcidin agonists, including synthetic peptides and small molecules that enhance hepcidin activity, represent promising approaches for treating iron overload disorders such as hereditary hemochromatosis and in patients on regular blood transfusions (3). Conversely, hepcidin antagonists have been developed to treat iron-restrictive anemias including ACD and certain cancer-related anemias (24). These approaches include anti-hepcidin antibodies, hepcidin-sequestering agents, and molecules that interfere with hepcidin-ferroportin binding (23). Clinical development of hepcidin modulators has faced challenges related to the complexity of iron regulation and potential off-target effects (25). The pleiotropic nature of iron metabolism requires careful consideration of dosing, timing, and patient selection to achieve therapeutic efficacy while avoiding adverse consequences (24).

Despite the compelling rationale for hepcidin modulation, significant controversies and challenges remain. The long-term consequences of therapeutic hepcidin suppression, a strategy for anemia of inflammation, are not fully known. Hepcidin is a key component of the body’s innate immune response to infection, as it sequesters iron from circulating pathogens. Chronic suppression could increase susceptibility to certain infections, a risk that requires careful long-term monitoring (25). Similarly, hepcidin agonists for iron overload must be carefully dosed to avoid iatrogenic iron-restricted erythropoiesis and anemia.

#### Anti-matriptase-2 antibodies

The development of anti-matriptase-2 antibodies represents a novel therapeutic approach for treating iron overload diseases by enhancing hepcidin expression (26). These fully humanized monoclonal antibodies target the enzymatic activity of matriptase-2, leading to increased hepcidin mRNA expression and decreased serum iron and transferrin saturation (27).

KY1066, a first-in-class antibody targeting matriptase-2, has been developed for the treatment of beta-thalassemia and other iron overload conditions (27). Preclinical studies demonstrated that reducing TMPRSS6 expression increases hepcidin expression, correcting iron overload, splenomegaly, and anemia in thalassemic mouse models (26). The pharmacokinetic and pharmacodynamic profiles of anti-matriptase-2 antibodies support their potential as best-in-class therapies for iron overload diseases (27).

#### Luspatercept and erythropoietic modulators

Luspatercept represents a breakthrough therapeutic approach for treating anemia in conditions characterized by ineffective erythropoiesis (28). Luspatercept is a first-in-class recombinant fusion protein that functions as a “ligand trap” for members of the transforming growth factor-β (TGF-β) superfamily, particularly activins and growth differentiation factor-11 (GDF-11). In healthy erythropoiesis, these signaling molecules act as brakes, inhibiting the late-stage maturation of erythroid precursors in the bone marrow. By binding and sequestering these inhibitory ligands, luspatercept releases the brake, promoting the development of mature, enucleated red blood cells (29–31).

Clinical trials have demonstrated significant efficacy of luspatercept in transfusion-dependent beta-thalassemia, with the BELIEVE trial showing a 21.4% reduction in transfusion burden compared to 4.5% with placebo (32). In myelodysplastic syndromes, the MEDALIST trial demonstrated that 38% of patients achieved transfusion independence for at least 8 weeks compared to 13% with placebo (33).

The COMMANDS trial, comparing luspatercept to erythropoiesis-stimulating agents in treatment-naive MDS patients, showed superior efficacy with 60.4% versus 34.8% achieving the primary endpoint of red blood cell transfusion independence (34). In a study evaluating luspatercept, a majority of patients achieved positive outcomes, with approximately 87% achieving transfusion independence (TI) for at least eight consecutive weeks within the first 24 weeks, a benefit largely driven by those with a low or moderate transfusion burden (LTB/MTB). Even among patients who were transfusion-dependent at baseline, 64% achieved a major hemoglobin increase. Despite the positive efficacy signals, the most common reason for treatment discontinuation was disease progression to higher-risk myelodysplastic syndromes (MDS), reported by about 45.5% of patients, though notably, no patients discontinued treatment due to adverse events (AEs) (30). Collectively, the BELIEVE, MEDALIST, and COMMANDS trials establish luspatercept as a transformative therapy for anemias driven by ineffective erythropoiesis. The consistent achievement of transfusion independence across different disease contexts (beta-thalassemia and MDS) validates the strategy of promoting erythroid maturation. However, as real-world evidence grows, challenges such as disease progression in MDS patients highlight that these agents are modulators of the disease phenotype rather than curative, underscoring the need for predictive biomarkers to identify the patients most likely to derive sustained benefit.

#### Anti-hemojuvelin approaches

Targeting hemojuvelin, the BMP6 co-receptor essential for iron-responsive hepcidin regulation, represents another promising therapeutic strategy (13). DISC-0974, a first-in-human anti-hemojuvelin monoclonal antibody, has been shown to reduce serum hepcidin levels and mobilize iron in healthy participants (35). A fully human anti-BMP6 antibody (KY1070) effectively counters the hepcidin-driven iron limitation characteristic of ACD by mobilizing endogenous iron deposits for red blood cell production. The combination of this antibody with erythropoietin-stimulating agents (ESA) showed a synergistic effect, significantly boosting the erythroid response and leading to a crucial EPO-sparing effect in rodent models of ACD (36).

#### JAK-STAT inhibition and hepcidin modulation: the momelotinib paradigm

The approval of momelotinib for the treatment of myelofibrosis represents a paradigm shift, demonstrating that hepcidin modulation can be a primary mechanism of action for a drug in a non-iron-overload disease. Momelotinib is a JAK1/JAK2 inhibitor that also directly inhibits ACVR1/ALK2, a receptor in the BMP signaling pathway (37). By attenuating BMP-SMAD signaling in hepatocytes, momelotinib reduces hepcidin production. This reduction in hepcidin mobilizes sequestered iron, making it available for erythropoiesis and directly correcting the anemia characteristic of myelofibrosis. The clinical success of momelotinib validates the BMP-hepcidin axis as a druggable target and provides a proof-of-concept for treating anemia by targeting upstream regulators of hepcidin transcription (37). Table 1 gives a summary of Iron metabolism modulators.

**TABLE 1:** Summary of iron metabolism modulators.

Agent type	Mechanism of action	Target condition(s)	Example agents/approaches	Key details and status
**Iron metabolism modulators: therapeutic approaches**				
Hepcidin agonists	Increase hepcidin activity or its signaling to reduce iron absorption and release.	Thalassemia, hereditary hemochromatosis, polycythemia vera.	TMPRSS6-targeting therapies: anti-matriptase-2 mAbs (e.g., KY1066); TMPRSS6 ASOs/SiRNAs	Preclinical/early clinical. reduces TMPRSS6 to increase endogenous hepcidin, correcting iron overload in animal models.
			Synthetic hepcidin mimetics: PTG-300; minihepcidins	Clinical trials. PTG-300 is a synthetic hepcidin analog shown in trials to reduce serum iron and transferrin saturation.
			BMP-signal enhancers: IMR-261 (NRF2 activator)	Preclinical. Activates the BMP6/SMAD pathway to stimulate hepcidin synthesis in the liver.
Hepcidin antagonists	Inhibit hepcidin’s function or production to increase iron availability for erythropoiesis.	Anemia of inflammation, chronic kidney disease, IRIDA.	Hepcidin-binding agents: spiegelmers (NOX-94); anticalins (PRS-080); anti-hepcidin mAbs (LY2787106)	Clinical trials. These molecules sequester circulating hepcidin, leading to increased serum iron and transferrin saturation in early-phase studies.
			Ferroportin stabilizers: VIT-2763	Clinical trials. A first-in-class oral agent that binds to ferroportin, preventing its hepcidin-mediated internalization.
			Hemojuvelin (HJV) inhibitors: DISC-0974 (anti-HJV mAb)	Clinical trials. Blocks the BMP co-receptor HJV to suppress hepcidin transcription, mobilizing iron for erythropoiesis.

## Ferroptosis as a therapeutic target

The dependence of hematologic malignancies on iron for proliferation and metabolism, as discussed in the context of systemic regulation, presents a unique therapeutic vulnerability. The discovery of ferroptosis–an iron-dependent form of programmed cell death driven by lipid peroxidation–has provided a mechanistic framework for exploiting this vulnerability. By strategically inducing this process, it is possible to target malignant cells that are often resistant to conventional apoptosis-based therapies (38, 39).

### Molecular mechanisms of ferroptosis

Ferroptosis is an iron-dependent form of programmed cell death characterized by excessive accumulation of lipid peroxides and reactive oxygen species (40). This distinct cell death pathway differs from apoptosis, necroptosis, and autophagy through its reliance on iron-catalyzed lipid peroxidation and sensitivity to iron chelation (41). The molecular machinery of ferroptosis involves three critical components: iron metabolism dysregulation, lipid peroxidation, and compromised antioxidant systems (39).

The ferroptosis pathway is primarily regulated by the glutathione peroxidase 4 (GPX4) antioxidant system, which normally prevents lipid peroxidation through the reduction of phospholipid hydroperoxides (38). Inactivation of GPX4 or depletion of its cofactor glutathione leads to accumulation of lipid peroxides, particularly in polyunsaturated fatty acid-rich membrane phospholipids (42). Iron serves as a catalyst in this process through the Fenton reaction, generating hydroxyl radicals that initiate and propagate lipid peroxidation (43).

#### Ferroptosis in hematologic malignancies

Hematologic malignancies demonstrate susceptibility to ferroptosis induction, making this pathway an attractive therapeutic target (44). A recent review highlighted the role of ferroptosis and its critical regulation by the GPX4/GSH antioxidant system. The authors confirm that tumor cells in various hematologic malignancies such as leukemia, lymphoma, and multiple myeloma are highly sensitive to this process. Manipulating these molecular mechanisms to induce ferroptosis presents a novel and promising therapeutic strategy for treating these often drug-resistant blood cancers (38).

While defective ferroptosis pathways can foster tumor development and therapy resistance, strategically inducing it offers a powerful therapeutic avenue; this is being pursued through novel agents such as System Xc^–^ inhibitors (e.g., Erastin), GPX4 inhibitors (e.g., RSL3), iron-based nanomaterials for targeted delivery, and combination regimens with conventional treatments, with emerging evidence also highlighting the potential of Traditional Chinese Medicine compounds like artemisinin to trigger this lethal process (42, 43, 45, 46).

### Therapeutic strategies targeting ferroptosis

The therapeutic modulation of ferroptosis in hematologic disorders is fundamentally context-dependent, pivoting on the paradoxical role of iron as an indispensable driver of the process and a primary pharmacological target. This duality defines a unique therapeutic landscape: while agents like erastin and RSL3 are designed to induce ferroptosis in malignant cells, traditional iron chelators can conversely modulate the process. This insight has catalyzed the development of innovative combination therapies that co-target iron availability and lipid peroxidation pathways, demonstrating enhanced anti-tumor efficacy in preclinical models. The successful clinical translation of these strategies, however, is contingent upon overcoming tumor heterogeneity and necessitates the concomitant development of robust predictive biomarkers to guide patient-specific treatment (38, 39, 43, 44, 46).

The systemic induction of ferroptosis presents a significant safety challenge. While the goal is to selectively target malignant cells, the fundamental reliance on iron and lipid peroxidation means that non-malignant tissues could also be affected, leading to potential off-target toxicity in organs such as the liver, kidney, and heart. The development of tumor-specific delivery systems (e.g., nanoparticle carriers) and reliable biomarkers to monitor ferroptosis *in vivo* is therefore paramount to the safe clinical translation of these strategies (39, 44). Table 2 shows various therapeutic strategies targeting Ferroptosis in hematologic malignancies.

**TABLE 2:** Ferroptosis as a therapeutic target in hematologic malignancies.

Strategy	Mechanism	Example agents/approaches
System Xc^–^ inhibition	Depletes intracellular glutathione by blocking cystine uptake.	Erastin, Sorafenib, Sulfasalazine.
GPX4 inhibition	Directly inactivates the key enzyme that repairs lipid peroxidation.	RSL3, ML162, (1S,3R)-ML210.
Iron delivery/modulation	Increases labile iron pool to catalyze lipid peroxidation; or uses chelators to protect non-malignant cells.	Iron-loaded nanoparticles, artemisinin derivatives; iron chelators (context-dependent).
Combination therapies	Induces ferroptosis while overcoming conventional drug resistance.	

## Iron chelation therapy: clinical applications and innovations

### Deferasirox: mechanisms and clinical outcomes

Deferasirox, a once-daily oral iron chelator, has revolutionized iron overload management in transfusion-dependent patients through its convenient dosing and demonstrated efficacy (47). The drug functions through selective iron chelation, forming stable complexes that are eliminated through biliary excretion (48).

Clinical studies have demonstrated significant reductions in labile plasma iron levels within hours of administration, with sustained decreases in iron burden over extended treatment periods (49).

The ESCALATOR study, evaluating deferasirox in heavily iron-overloaded beta-thalassemia patients, demonstrated clinically meaningful reductions in liver iron concentration and serum ferritin levels (49). Patients with baseline liver iron concentrations exceeding 7 mg/g dry weight achieved significant iron removal with deferasirox doses of 20–30 mg/kg daily (48).

The EPIC study, involving 1,744 patients with various transfusion-dependent anemias, confirmed the efficacy of tailored deferasirox dosing based on transfusional iron intake and serum ferritin trends (50).

### Combination iron chelation strategies

The development of combination iron chelation regimens has addressed the limitations of monotherapy in patients with severe iron overload or organ-specific complications (51).

The combination of deferasirox and deferoxamine has demonstrated superior iron removal compared to either agent alone, with particular benefits in cardiac iron clearance (52).

A pilot clinical trial of combined deferasirox and deferoxamine therapy showed a 31% reduction in median liver iron concentration over 12 months (51).

Triple combination therapy incorporating deferasirox, deferiprone, and deferoxamine is being evaluated for patients with very high iron overload who fail to respond adequately to dual chelation (53). The synergistic effects of combining different chelation mechanisms–deferasirox targeting intracellular iron, deferiprone providing cardiac protection, and deferoxamine offering potent iron removal–may optimize therapeutic outcomes (52).

Real-world studies have confirmed the safety and efficacy of combination chelation in patients with myelodysplastic syndromes and other transfusion-dependent conditions (54).

#### Novel applications and mechanisms

Emerging clinical evidence indicates that the therapeutic utility of deferasirox extends beyond its conventional application for iron overload, revealing benefits in hematologic disorders not exclusively attributable to iron burden reduction. Notably, a case report in primary myelofibrosis, documents the correction of anemia and attainment of transfusion independence following deferasirox administration, implying pleiotropic mechanisms of action beyond mere iron chelation (55). This concept of synergistic benefit is further supported by findings that combining deferasirox with hydroxyurea in sickle cell disease yields enhanced clinical outcomes surpassing monotherapy with either agent (56). The pharmacological profile of deferasirox is also influenced by individual genetic makeup; pharmacogenomic studies have identified variants in genes such as PON-1 and UGT1A1 that modulate treatment efficacy and impact ancillary mineral metabolism, including zinc homeostasis (57). Concurrently, pharmaceutical advancements have improved the clinical implementation of chelation therapy through the development of novel formulations, such as granule preparations, which enhance patient compliance and tolerability while preserving therapeutic efficacy compared to traditional dispersible tablets (58). These developments, coupled with refined age-specific treatment protocols, have facilitated the safe and evidence-based initiation of iron chelation in pediatric cohorts, including children as young as 2 years of age (59).

## Clinical controversies and challenges

### Patient selection and biomarker development

The optimal stratification of patients for iron-targeted therapies presents a considerable clinical challenge, particularly in pathologies characterized by dysregulated iron metabolism rather than systemic overload alone (60). This is evidenced by real-world data in elderly patients with low-risk myelodysplastic syndrome, which reveals substantial heterogeneity in treatment application and clinical outcomes (60). Advancing precision medicine in this domain is contingent upon the development of validated predictive biomarkers. For hematologic malignancies, biomarkers of ferroptosis sensitivity are a promising avenue, though they require extensive validation (39). Concurrently, distinct iron dysregulation signatures in pediatric leukemia, involving proteins such as matriptase-2 and neogenin-1, offer potential as novel biomarkers for therapeutic stratification (61). Given the complexity of iron homeostasis, future diagnostics will likely rely on comprehensive biomarker panels rather than single-parameter assessments to accurately capture the underlying pathophysiology (15). In this context, growth differentiation factor-15 (GDF-15) has emerged as a promising biomarker for monitoring iron metabolism disorders and treatment response (62).

### Safety considerations and long-term outcomes

The surveillance of novel iron-targeted therapies remains imperative, especially for agents with limited long-term clinical data (32). While treatments such as luspatercept have demonstrated sustained efficacy and a manageable safety profile over 5 years in patients with beta-thalassemia (63), the long-term immunological consequences of chronic hepcidin modulation, including its impact on infection susceptibility, are not yet fully elucidated (25). Therapy in vulnerable populations, including patients with pre-existing renal or cardiac impairment, necessitates vigilant monitoring and potential dose adjustment (64). Specific agent-related toxicities, such as deferasirox-induced nephrotoxicity and proximal tubulopathy, have prompted the investigation of renoprotective strategies, including adjunctive vitamin E supplementation (64). Finally, ensuring long-term adherence to oral iron chelation regimens remains a pervasive challenge, underscoring the importance of developing improved drug formulations and robust patient education strategies to enhance compliance (58).

## Future directions and research priorities

### Precision medicine approaches

The convergence of pharmacogenomic data with established iron metabolism biomarkers holds considerable promise for advancing personalized therapeutic strategies for iron-related disorders (21). Specific genetic polymorphisms, such as those in TMPRSS6 which modulate response to oral iron, may serve as predictive biomarkers for the efficacy of emerging therapies, including anti-matriptase-2 antibodies (21). This paradigm supports the development of comprehensive genetic panels that interrogate key iron-regulatory genes to inform precise treatment selection and dosing regimens (57). Beyond discrete genetic markers, artificial intelligence and machine learning approaches are poised to decipher complex, multi-factorial biomarker signatures capable of predicting therapeutic response across a spectrum of hematologic diseases (30). Furthermore, the systematic integration of multi-omics data–spanning transcriptomics, proteomics, and metabolomics–promises to elucidate previously uncharacterized nodes within iron regulatory networks, thereby revealing novel therapeutic targets (61). Complementing these analytical approaches, patient-derived cellular models and organoid systems provide a physiologically relevant platform for *ex vivo* drug screening and the optimization of treatment protocols on an individualized basis (46).

### Novel therapeutic targets and combination strategies

The therapeutic landscape for hematologic disorders is being reshaped by the identification of novel targets within iron regulatory pathways, expanding the arsenal of available treatments (3). This includes the development of small-molecule inhibitors that precisely engage specific components of the ferroptosis pathway to induce selective cell death in malignant cells (46), as well as NRF2 activators that modulate iron homeostasis by augmenting BMP6-mediated hepcidin synthesis for the treatment of iron overload (65). To maximize therapeutic impact, combination strategies that concurrently target multiple nodes of iron metabolism are under investigation, aiming for superior efficacy and a reduced toxicity profile (66). Furthermore, the integration of these iron-targeted modalities with established immunotherapies and cellular therapies constitutes a burgeoning frontier in oncology clinical research (45). The application of nanotechnology offers a parallel advancement, with engineered delivery systems designed to enhance the specificity and therapeutic index of iron modulators by minimizing off-target effects (46).

### Translational research opportunities

The translation of these mechanistic insights into clinical practice is contingent upon robust translational research infrastructure (41). Key opportunities include the refinement of advanced imaging techniques to monitor tissue-specific iron distribution for precise treatment guidance (67) and the development of non-invasive biomarkers to quantify ferroptosis activity *in vivo* (39). Success in this domain, particularly for rare hematologic disorders, will require international collaboration to power definitive clinical trials (53). The establishment of comprehensive patient registries and linked biobanks will be instrumental in accelerating discovery and enabling long-term outcome surveillance (60). Finally, the implementation of targeted educational initiatives for clinicians is paramount to ensuring the optimal application of these complex therapies, thereby improving patient outcomes and mitigating adverse events (47).

## Conclusion

In conclusion, the therapeutic landscape for hematologic disorders is being fundamentally reshaped by advanced understanding of iron homeostasis. Targeting the hepcidin-ferroportin axis with novel agonists and antagonists offers a powerful strategy to correct iron dysregulation in both overload and deficiency states. Simultaneously, the strategic induction of ferroptosis presents a promising avenue for eliminating malignant cells in hematologic cancers. The success of erythroid maturation agents like luspatercept further underscores the therapeutic potential of modulating erythropoiesis. The future of this field lies in precision medicine, leveraging pharmacogenomics and biomarker-driven approaches to optimize these sophisticated therapies. Overcoming challenges related to patient stratification, long-term safety, and clinical translation will be crucial to fully realizing the potential of these groundbreaking treatments and improving outcomes for patients with complex hematologic diseases.
